# QuantiFERON-TB reversion in children and adolescents with tuberculosis

**DOI:** 10.3389/fimmu.2024.1310472

**Published:** 2024-03-21

**Authors:** Paula Rodríguez-Molino, Araceli González Sánchez, Antoni Noguera-Julián, Aleix Soler-García, Patricia Martínez Paz, Ana Méndez-Echevarría, Fernando Baquero-Artigao, Miguel González Muñoz, María Jesús Ruíz-Serrano, Manuel Monsonís, Rocío Sánchez León, Jesús Saavedra-Lozano, Begoña Santiago-García, Talía Sainz

**Affiliations:** ^1^ General Pediatrics, Infectious and Tropical Diseases Department, Hospital La Paz, Madrid, Spain; ^2^ La Paz Research Institute (IdiPAZ), Madrid, Spain; ^3^ Universidad Autónoma de Madrid (UAM), Madrid, Spain; ^4^ Centro de Investigación Biomédica en Red en Enfermedades Infecciosas (CIBERINFEC), Madrid, Spain; ^5^ General Pediatrics, Hospital Puerta de Hierro, Madrid, Spain; ^6^ Malalties Infeccioses i Resposta Inflamatòria Sistèmica en Pediatria, Servei de Malalties Infeccioses Patologia Importada, Institut de Recerca Pediàtrica Sant Joan de Déu, Barcelona, Spain; ^7^ Departament de Cirurgia i Especialitats Medicoquirúrgiques, Facultat de Medicina i Ciències de la Salut, Universitat de Barcelona, Barcelona, Spain; ^8^ Centro de Investigación Biomédica en Red de Epidemiología y Salud Pública, Instituto de Salud Carlos III, Madrid, Spain; ^9^ Red de Investigación Translacional en Infectología Pediátrica (RITIP), Madrid, Spain; ^10^ Department of Immunology, Hospital La Paz, Madrid, Spain; ^11^ Clinical Microbiology and Infectious Diseases, Hospital General Universitario Gregorio Marañon, Madrid, Spain; ^12^ Instituto de Investigación Sanitaria Gregorio Marañón (IiSGM), Madrid, Spain; ^13^ Centro de Investigación Biomédica en Red (CIBER) de Enfermedades Respiratorias - CIBERES, Madrid, Spain; ^14^ Servei de Microbiologia, Hospital Sant Joan de Déu, Barcelona, Spain; ^15^ Infectious Diseases Department, General Pediatrics, Hospital Gregorio Marañón, Madrid, Spain

**Keywords:** QuantiFERON-TB, reversion rate, tuberculosis, children, adolescents

## Abstract

We analyzed 136 children with tuberculosis disease or infection and a positive QuantiFERON-TB (QFT) assay, followed-up for a median of 21 months (0.4-11years). QFT reversed in 16.9% of cases, with significant decreases in TB1 (-1.72 *vs*. -0.03 IU/ml, p=0.001) and TB2 (-1.65 *vs*. -0.43 IU/ml, p=0.005) levels compared to non-reverters. We found a higher QFT reversion rate among children under 5 years (25.0% *vs* 11.9%, p=0.042), and those with TST induration <15mm (29% *vs* 13.3%, p=0.055). Our data reveal that, although QFT test remained positive in the majority of children, reversion occurred in 16% of cases in a progressive and stable pattern. Younger age and reduced TST induration were associated with QFT reversion.

## Introduction

Tuberculosis (TB) remains a pressing global health concern, with children accounting for an estimated 10-20% of the annual cases of TB. In 2021, Europe reported 6,556 new TB cases in children (<15 years), comprising 4% of the total burden (1). While Spain is classified as a low TB burden country with 6.9 cases per 100,000 inhabitants in 2022, it has the highest TB incidence among European countries of similar economic status, second only to Portugal (2).

Children are at heightened risk of severe and disseminated tuberculosis (TB) forms. However, microbiological confirmation occurs in less than half of cases, and the diagnosis often relies on a combination of clinical and radiological findings, epidemiological risk factors, and a positive tuberculin skin test (TST) or interferon-γ (IFN-γ) release assay (IGRA) result (3).

While TST is known to remain positive, limited data are available regarding the medium-term evolution of QuantiFERON-TB (QFT) (4). Various factors, including host-related, environmental and technical aspects, can influence QFT results, which cannot distinguish reinfection or reactivation (5).

In adults, studies addressing antituberculosis treatment’s effect on QFT results has yielded conflicting findings (6) with reversal rates spanning from 32% to 62% (7, 8). A South African study in infants with TB disease (<6 months old) reported a 58% reversal rate during a 6 to 24-month follow-up (9). Based on current evidence, guidelines do not recommend assessing QTF reversion as a parameter for monitoring treatment response (3), nor in cases of TB contact with an initial positive result to reduce the number of preventive treatments (10). Pediatric data are scarce, mainly restricted to the treatment period (11), and the influence of age remains uncertain (12).

Our study aims to assess the long-term dynamics of QFT in children with TB infection (TBI) or TB disease (TBD), providing insights for clinicians facing reinfection/reactivation challenges, particularly in this vulnerable population.

## Methods

We conducted a multi-center longitudinal study involving children and adolescents under 18 years of age diagnosed with either TBI or TBD with a positive QFT test (QFT-TB Gold-In-Tube/Gold-In-Tube-PLUS; Qiagen, Venlo, Netherlands). The study spanned from March 2011 to May 2023 and took place at three tertiary hospitals in Spain. We actively contacted all patients with only one positive QFT test performed to offer a second determination, and subsequently included them in the study. Clinical data were extracted from medical records, and we gathered epidemiological factors such as visits to high-prevalence countries and/or known TB contact post-treatment completion.

We adhered to established consensus criteria for defining TB disease (13). Patients with signs/symptoms of TB and a microbiological confirmation by culture and/or PCR were classified as *Confirmed TB*. *Unconfirmed TB* was defined as a patient lacking microbiological confirmation but meeting at least two of the following criteria: (a) symptoms suggestive of TB, (b) chest radiograph consistent with TB, (c) known TB exposure or immunologic evidence of *M. tuberculosis* infection and (d) response to TB treatment.

The study received approval from the Ethical and Research Committee of Hospital Universitario La Paz (PI-5431) and participating centers. Written informed consent was obtained from all parents/guardians and also from children aged ≥12 years.

Continuous variables were described using medians and interquartile ranges (IQR), while categorical variables were expressed as absolute and relative frequencies. A positive QFT was defined as a TB antigen – Nil IFN-γ value greater than or equal to 0.35 IU/ml, following the manufacturer’s guidelines. The assessment of QFT dynamics involved two approaches: firstly, by comparing the cumulative rate of QFT reversions over time, and secondly, by using the delta value to quantify changes in QFT quantitative values for TB1 and TB2. For this report, TB1 and TB2 IFN-γ concentrations (in IU/ml) are background-corrected (IFN-γ concentration in Antigen-stimulated minus Nil tube concentration). Statistical analyses were performed using STATA v17 and/or Prism 9.0.

## Results

A total of 136 children and adolescents were enrolled, including 50 with TBI and 86 with TBD. Diagnosis through contact tracing was conducted in 83 patients (45 TBD and 38 TBI). Among the TBD group, 31 (36%) received microbiological confirmation, with 19 of them (61.3%) being under 5 years old. The median age at diagnosis was 5.8 years [3.2-11.3], with 40% of females. Only two patients had immunosuppressive conditions, one with systemic sclerosis and another with severe malnutrition. All participants initiated treatment according to local guidelines, successfully completed anti-TB therapy, and achieved favorable outcomes.

**Figure 1:**
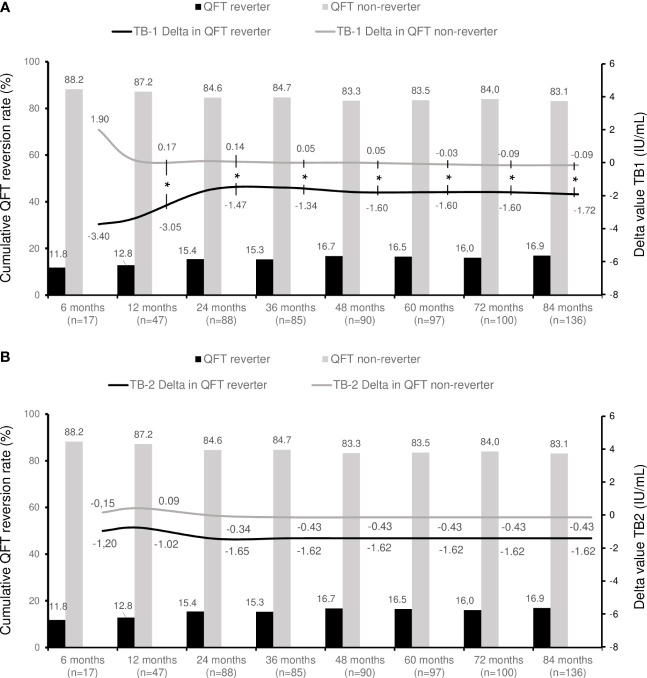
QuantiFERON dynamics over time in the study population. Bar chart represents the cumulative rate of QFT reversion during follow up. Lines depict the extent of change of TB1 **(A)** and TB2 **(B)** quantitative results using delta values for patients who revert QTF compared to those who do not (background-corrected with Nil tube). (QFT), QuantiFERON-TB. (n), number of patients. Distributions were no normal and comparisons were made with the Mann-Whitney’s t-test. *Statistically significant difference in delta values between patients with QFT reversal and those who do not.

The median interval to the second QFT determination was 21 months (range [3 months - 11 years]). QFT reversion occurred in 23 cases (16.9%). **Table 1** provides a summary of clinical and epidemiological characteristics based on QFT reversion, while **Figure 1** illustrates the cumulative rate of QFT reversion over time. Although the exact median time for QFT reversal was not predetermined, the cumulative rate consistently increased in the first 48 months, indicating an ongoing reversal process within the initial 7 years post-treatment.

**Table 1:** Baseline characteristics of the 136 children included in the study.

	All patients (n=136)	QFT reverters (n=23)	QFT non-reverters (n=113)	p value
Age years, median [IQR]	5.8 [3.2-11.3]	4.4 [2.4-11.2]	6.3 [3.6-11.3]	ns
<2 years	20 (14.7)	5 (21.7)	15 (13.3)	ns
2-5 years	50 (36.8)	10 (43.5)	40 (35.4)	ns
>5 years	66 (48.5)	8 (34.8)	58 (51.3)	ns
Sex female, n (%)	55 (40.4)	12 (52.2)	43 (38.1)	ns
Region of birth				ns
African Region	5 (3.7)	1 (4.4)	4 (3.5)	
Region of the Americas	16 (11.8)	3 (13)	13 (11.5)	
South-East Asia Region	3 (2.2)	0 (0)	3 (2.7)	
European Region	105 (77.2)	19 (82.6)	86 (76.1)	
Eastern Mediterranean Region	7 (5.1)	0 (0)	7 (6.2)	
Ethnic background				ns
White Caucasian	94 (69.1)	18 (78.3)	76 (67.3)	
Black/African American	8 (5.9)	1 (4.4)	7 (6.2)	
Latin American	22 (16.2)	4 (17.4)	18 (15.9)	
Asian	8 (5.9)	0 (0)	8 (7.1)	
Arab or Berber	3 (2.2)	0 (0)	3 (2.7)	
Mixed race	1 (0.7)	0 (0)	1 (0.9)	
Migrant status				
Born abroad	35 (25.7)	6 (26.1)	29 (25.7)	ns
Born in Spain	101 (74.3)	17 (73.9)	84 (74.3)	ns
Tuberculosis diagnosis				
TBI	50 (36.8)	11 (47.8)	39 (34.5)	ns
TBD	86 (63.2)	12 (52.2)	74 (65.5)	ns
Unconfirmed TB	55 (64)	10 (83.3)	45 (60.8)	ns
Confirmed TB	31 (36)	2 (16.7)	29 (39.2)	ns
Pulmonary	78 (90.7)	11 (73)	67 (78)	ns
Extrapulmonary	12 (14)	3 (25)	9 (16.4)	ns
Reported underlying condition (n=59)	23 (39)	5 (21.7)	18 (78.3)	ns
Prematurity	3 (13)	2 (40)	1 (5.6)	
Cardiovascular	2 (8.7)	1 (20)	1 (5.6)	
Respiratory	13 (56.5)	3 (60)	10 (55.6)	
Neurology	5 (21.7)	2 (40)	3 (16.7)	
Gastrointestinal	7 (30.4)	2 (40)	5 (27.8)	
Rheumatology	2 (8.7)	2 (40)	0 (0)	
Nephrology	0 (0)	0 (0)	0 (0)	
Atopic dermatitis	2 (8.7)	0 (0)	2 (11.1)	
Haematological malignancy	0 (0)	0 (0)	0 (0)	
Solid organ malignancy	1 (4.3)	0 (0)	1 (5.6)	
Immune disorder	2 (8.7)	1 (20)	1 (5.6)	
Malnutrition	2 (8.7)	1 (20)	1 (5.6)	
BCG status (n=129)				
Yes	26 (20.2)	6 (23.1)	15 (14.6)	ns
No	103 (79.8)	20 (76.9)	88 (85.4)	ns

Data shown are numbers and percentages unless stated otherwise. IQR, interquartile range. QFT, QuantiFERON-TB. TBI, tuberculosis infection. TBD, tuberculosis disease. BCG, Bacillus Calmette–Guérin. Comparisons were made with the chi-square test.

ns, not significant.

Among children under 5 years, QFT reversion was notably higher (25.0% *vs* 11.9%, p=0.042). Although the median time to the second QFT determination was longer in TBD (27 months [10-94] *vs* 19.5 months [9-25]; p=0.09), QFT reversion was more common in individuals with TBI than in those with TBD, albeit not statistically significant (22% [11/50] *vs*. 14% [12/86]; p=0.227). No significant associations were found based on underlying disease, BCG vaccination, or migrant status. Limited data precluded the comparison of QFT reversion based on TB microbiological confirmation, though there was a trend towards lower reversion in confirmed TBD (16.7% *vs* 39.2%; p=0.197) (**Table 1**).

**Figure 2:**
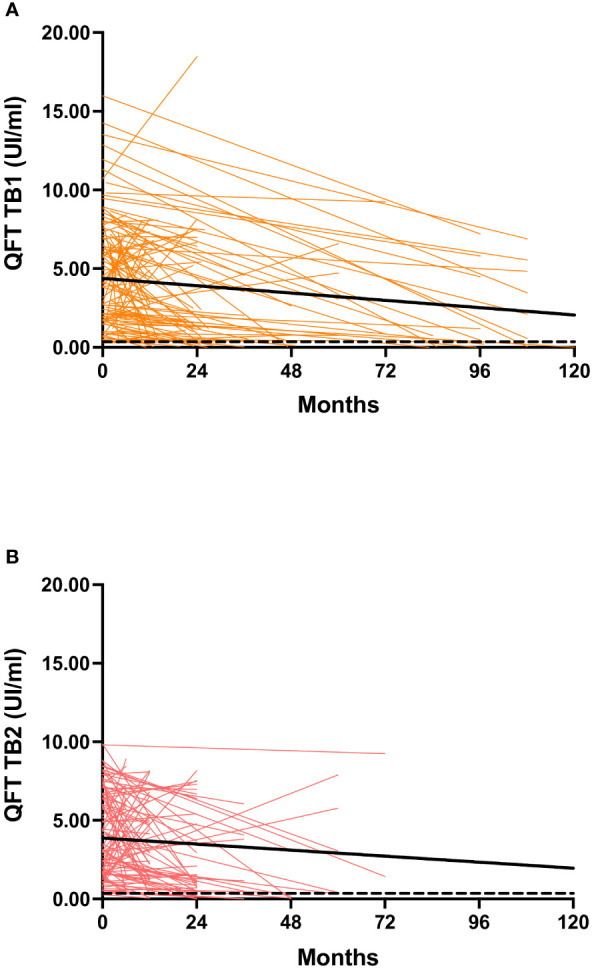
Background-corrected IFN-γ levels at baseline and during follow-up in the study population. **(A)** QFT-Plus TB1 tube; **(B)** QFT-Plus TB2 tube. Bold lines represent the medians; the dashed line indicates the threshold of positivity (0.35 IU/mL) according to the manufacturer. QFT: QuantiFERON-TB; IU: international units. Trendline was made using the least square regression method.

Throughout the longitudinal follow-up, a decline in quantitative values was observed in both TB1 and TB2 tubes, as indicated by a median delta value of -0.72 for TB1 [-3.6 to 0.57] and -0.70 for TB2 [-2.2 to 0.5] (**Figures 1**, **2**). Importantly, this decline was more pronounced among individuals with QFT reversion compared to those without reversion, both for TB1 tubes (-1.72 [-5.3 to -0.8] *vs* -0.03 [-1.77 to 2.31], p=0.001) and TB2 tubes (-1.65 [-4.08 to -0.42] *vs* -0.43 [-1.95 to 1.07], p=0.005).

The correlation between TB1 and TB2 was high (Spearman’s Rho 0.697, p < 0.001) and similar among children who reverted (Rho 0.776, p < 0.001) and those who did not revert (Rho 0.676, p < 0.001). No significant differences in QFT quantitative values were found based on TBI or TBD status.

TST results were available in 127 patients at diagnosis (93.4%), with 119 testing positive and a median induration of 17 mm [15-21]. There were no differences between TBI and TBD (17mm [13-20] *vs* 17mm [15-21]; p=0.557). Interestingly, among patients with TST>=15mm, we noted a trend toward a lower QFT reversion rate (13.3% *vs* 29%, p=0.055).

## Discussion

In this study, we present a series of children diagnosed with TB infection and TB disease, providing insights into the dynamics of QuantiFERON-TB reversion over a median follow-up of 21 months within a low-prevalence country. Our data reveal that the QFT test remains positive in the majority of children but transitions to negative in approximately 16% of cases. Notably, QFT reversion appears to follow a progressive and stable pattern over time, characterized by declining absolute values for both TB1 and TB2 measurements. While younger age and reduced TST induration were associated with QFT reversion, TBI/TBD status did not show a significant correlation.

The role of QFT in monitoring TB treatment remains an active research area. However, existing longitudinal data primarily focused on adults during post-treatment phases have yielded conflicting findings. Consistent with our study, previous pediatric series have reported QFT reversion rates ranging from 15% to 16.9% (11, 14), in contrast to adult series where rates vary from 32% to 62% (7, 8). Age-related differences in QFT reversion can be attributed to factors such as immune aging, comorbidities, chronic illnesses, and immunosuppressive therapies. Notably, our findings indicate a higher reversion rate among children younger than 5 years, suggesting that immune response fluctuations at the extremes of age contribute to variations in QFT results and a higher likelihood of reversion. In contrast to a study in South Africa involving infants (9), we did not observe a heightened reversion among children under 2 years, likely due to our limited sample size. Based on the current evidence, guidelines do not recommend assessing QTF reversion for treatment response monitoring (15). Vigilance in excluding TB is crucial when suspecting TB reinfection or reactivation, even if QFT reverts, as it does not definitively indicate disease absence or resolution.

Children with TBI showed a higher reversion rate than those with TBD, though not statistically significant, potentially attributed to diminished mycobacterial antigen exposure and a less robust cytokine response. In contrast, patients with greater TST induration had higher TB1 and TB2 values, and lower reversal rates, suggesting that eliciting a potent immune response after infection leads to a more prolonged response. These results are in line with other studies that describe that children with baseline positive TST were less likely to have QFT reversion (AOR 0.01 95%CI [0.001-0.24]) (14).

Interestingly, our study did not record any indeterminate QFT results in the follow-up assays. This could be attributed to the smaller number of children under 5 or those with immunosuppression in our cohort (3). Although the reasons for indeterminate QFT results are not fully understood, some authors have suggested that the quality, not just the quantity, of the immune response may contribute to indeterminate outcomes.

Our study does have several limitations. Firstly, we lack multiple QFT test determinations over time for each patient, precluding precise timing identification for reversion cases. To address this limitation, we grouped patients by different timepoints. Secondly, in our cohort, we cannot determine whether the test reversion is sustained over time or temporary. Thirdly, we employed two different QFT test versions (QFT-TB Gold In Tube/Gold In Tube PLUS); however, recent literature indicates no significant sensitivity and specificity differences between these tests (3). Lastly, our study was conducted in a low TB prevalence country with a limited risk of reinfection, potentially limiting its applicability to regions with high TB/HIV burdens. The subanalysis for immunocompromised children and those living with HIV was insufficient due to group size.

## Conclusions

In summary, our study highlights the occurrence of progressive QFT reversion over time in a notable percentage of children. Larger studies are essential to delve deeper into the factors influencing QFT reversion in children and to explore its evolution beyond the first 5 years following exposure. This information is crucial for guiding clinicians in assessing QFT’s performance in suspected reinfections/reactivations, particularly in cases of immunosuppression, which is an increasingly prevalent situation in both high and low-income countries.

## Data availability statement

The original contributions presented in the study are included in the article/supplementary material. Further inquiries can be directed to the corresponding author.

## Ethics statement

The studies involving humans were approved by Ethical and Research Committee of Hospital Universitario La Paz (PI-5431). The studies were conducted in accordance with the local legislation and institutional requirements. Written informed consent for participation in this study was provided by the participants’ legal guardians/next of kin.

## Author contributions

PR-M: Writing – review & editing, Writing – original draft, Visualization, Software, Resources, Project administration, Methodology, Investigation, Funding acquisition, Data curation, Conceptualization. AG: Writing – review & editing, Methodology. AN-J: Writing – review & editing, Methodology. AS-G: Writing – review & editing, Methodology. PP: Writing – review & editing, Methodology. AM-E: Writing – review & editing, Conceptualization. FB-A: Writing – review & editing, Conceptualization. MGM: Writing – review & editing, Methodology. MR-S: Writing – review & editing, Methodology. MM: Writing – review & editing, Methodology. RS: Writing – review & editing, Methodology. JS-L: Writing – review & editing. BS-G: Writing – review & editing, Visualization, Supervision, Project administration, Methodology, Conceptualization. TS: Writing – review & editing, Supervision, Project administration, Methodology, Formal Analysis, Conceptualization.
